# Evidence from comprehensive independent validation studies for smooth pursuit dysfunction as a sensorimotor biomarker for psychosis

**DOI:** 10.1038/s41598-024-64487-6

**Published:** 2024-06-15

**Authors:** Inga Meyhoefer, Andreas Sprenger, David Derad, Dominik Grotegerd, Ramona Leenings, Elisabeth J. Leehr, Fabian Breuer, Marian Surmann, Karen Rolfes, Volker Arolt, Georg Romer, Markus Lappe, Johanna Rehder, Nikolaos Koutsouleris, Stefan Borgwardt, Frauke Schultze-Lutter, Eva Meisenzahl, Tilo T. J. Kircher, Sarah S. Keedy, Jeffrey R. Bishop, Elena I. Ivleva, Jennifer E. McDowell, James L. Reilly, Scot Kristian Hill, Godfrey D. Pearlson, Carol A. Tamminga, Matcheri S. Keshavan, Elliot S. Gershon, Brett A. Clementz, John A. Sweeney, Tim Hahn, Udo Dannlowski, Rebekka Lencer

**Affiliations:** 1 Institute for Translational Psychiatry, University of Muenster, Albert Schweitzer Campus 1, Build. A9a, 48149 Muenster, Germany; 2 Otto-Creutzfeldt Center for Cognitive and Behavioral Neuroscience, University of Muenster, Muenster, Germany; 3 Department of Psychiatry and Psychotherapy, Medical Faculty, Heinrich-Heine University, Duesseldorf/LVR, Duesseldorf, Germany; 4 Department of Neurology, University of Luebeck, Luebeck, Germany; 5 Department of Child Adolescence Psychiatry and Psychotherapy, University of Muenster, Muenster, Germany; 6 Institute of Psychology, University of Muenster, Muenster, Germany; 7 Department of Psychiatry and Psychotherapy, Ludwig-Maximilian University Munich, Munich, Germany; 8 Institute of Psychiatry, Psychology and Neuroscience, King’s College London, London, UK; 9 Max-Planck-Institute of Psychiatry Munich, Munich, Germany; 10 Department of Psychiatry and Psychotherapy, University of Luebeck, Luebeck, Germany; 11 Department of Psychiatry, Psychiatric University Hospital, University of Basel, Basel, Switzerland; 12 Department of Psychology, Faculty of Psychology, Airlangga University, Surabaya, Indonesia; 13 University Hospital of Child and Adolescent Psychiatry and Psychotherapy, University of Bern, Bern, Switzerland; 14 Department of Psychiatry and Psychotherapy, Philipps-University Marburg, Marburg, Germany; 15 Department of Psychiatry and Behavioral Neuroscience, University of Chicago, Chicago, USA; 16 Department of Experimental and Clinical Pharmacology and Department of Psychiatry and Behavioral Sciences, University of Minnesota, Minneapolis, USA; 17 Department of Psychiatry, The University of Texas Southwestern Medical Center, Dallas, TX USA; 18 Departments of Psychology and Neuroscience, Bio-Imaging Research Center, University of Georgia, Athens, GA USA; 19 Department of Psychiatry and Behavioral Sciences, Northwestern University Feinberg School of Medicine, Chicago, IL USA; 20 Department of Psychology, Rosalind Franklin University of Medicine and Science, Chicago, IL USA; 21 Departments of Psychiatry and Neuroscience, Yale School of Medicine, and Olin Research Center, Institute of Living/Hartford Hospital, Hartford, CT USA; 22 Department of Psychiatry, Harvard Medical School, Beth Israel Deaconess Medical Center, Boston, MA USA; 23 Huaxi MR Research Center (HMRRC), Department of Radiology, West China Hospital of Sichuan University, Chengdu, China; 24 Department of Psychiatry and Behavioral Neuroscience, University of Cincinnati College of Medicine, Cincinnati, USA

**Keywords:** Smooth pursuit eye movements, Machine learning, Individual prediction, Psychosis, Bipolar, Depression, Neuroscience, Psychology, Biomarkers, Diseases, Neurology

## Abstract

Smooth pursuit eye movements are considered a well-established and quantifiable biomarker of sensorimotor function in psychosis research. Identifying psychotic syndromes on an individual level based on neurobiological markers is limited by heterogeneity and requires comprehensive external validation to avoid overestimation of prediction models. Here, we studied quantifiable sensorimotor measures derived from smooth pursuit eye movements in a large sample of psychosis probands (N = 674) and healthy controls (N = 305) using multivariate pattern analysis. Balanced accuracies of 64% for the prediction of psychosis status are in line with recent results from other large heterogenous psychiatric samples. They are confirmed by external validation in independent large samples including probands with (1) psychosis (N = 727) versus healthy controls (N = 292), (2) psychotic (N = 49) and non-psychotic bipolar disorder (N = 36), and (3) non-psychotic affective disorders (N = 119) and psychosis (N = 51) yielding accuracies of 65%, 66% and 58%, respectively, albeit slightly different psychosis syndromes. Our findings make a significant contribution to the identification of biologically defined profiles of heterogeneous psychosis syndromes on an individual level underlining the impact of sensorimotor dysfunction in psychosis.

## Introduction

Given the generally weak associations between clinically defined psychiatric diagnoses with specific neurobiological alterations of the central nervous system, the development and validation of biomarkers has been a major goal in psychiatric research for decades^1^. Many studies have combined a large number of variables and/or multiple biomarkers using multivariate pattern recognition approaches^2–9^. There is growing interest in parameters affecting the stability of these results including internal and external validation procedures as well as sample sizes of training and validation samples as validation sample size must be regarded as major risk of misestimation^10–13^. This finding might explain why larger data sets tend to display weaker (presumable closer to “true”) accuracies (e.g. in the classification of depressive patients vs. healthy controls 60–65% accuracy based on structural MRI data in N = 2240 participants^11^ or 54–56% accuracy based on different neuroimaging modalities in N = 1809 participants^14^) than many previous findings in small samples (e.g.Refs.^15,16^).

One well-established and quantifiable biomarker in psychosis research is smooth pursuit eye movements (SPEM). SPEM testing involves having individuals visually track a small moving object relying on continuous sensorimotor processing of perceptual motion signals into dynamic adjustments of motor actions^17^. Thus, specific SPEM parameters reflect the ability of the brain to continuously receive visual motion information and simultaneously generate, monitor and adjust motor output accordingly to provide a clear visual percept of a moving object of interest. As early as 1908, numerous studies have emphasized SPEM dysfunctions as a biomarker for schizophrenia and other psychotic disorders indicating specific impairments of visual sensorimotor processing not only in stable but also early states of the disorder^18–26^.

The assessment of SPEM was recently included in studies initiated by the Bipolar-Schizophrenia Network on Intermediate Phenotypes (B-SNIP) consortium aiming to develop a biologically valid framework (e.g. biologically defined phenotypes) for psychotic disorders (i.e. stable probands with schizophrenia, schizoaffective disorder, or psychotic bipolar-I disorder)^9,27–30^. With regard to psychosis symptoms in the B-SNIP1 sample, Reininghaus and colleagues^31^ reported evidence of a transdiagnostic dimension underlying affective and non-affective psychotic symptoms. In line with this, results from the first recruitment period of the B-SNIP study (B-SNIP1, N = 674) indicate SPEM deterioration not only in schizophrenia but also in probands with schizoaffective and bipolar disorder with psychotic symptoms^20^. These findings, consistent with smaller sample studies^25^, imply that SPEM deficits can be regarded as a transdiagnostic biomarker for psychosis.

To determine the specificity of the relationship between psychotic symptoms and SPEM performance, it is essential to study probands with disorders that lack psychotic symptoms such as non-psychotic affective, substance, attention-deficit/hyperactivity, and obsessive–compulsive. Such studies revealed either intact SPEM performance or only minimal SPEM deficits^32–36^. As sample sizes were rather small for most of these studies, however, conclusions remain unclear. Underlining its usefulness as a biomarker, subtle SPEM deficits were not only found in chronically ill but also in first episode patients^23,37,38^ and in unaffected first-degree relatives of schizophrenia patients^20,39^. Such subtle SPEM deficits are reflected by specific impairments of certain SPEM measures, e.g. during SPEM initiation, while other SPEM measures, e.g. sustained eye velocity, appear unimpaired indicating that certain compensation mechanisms within the oculomotor systems, e.g. derived from prediction, are in play^20,40^. Thus, we would expect that similar SPEM disturbances would also be present in a clinical high-risk state for psychosis^41^ which has not been investigated so far.

**Figure 1:**
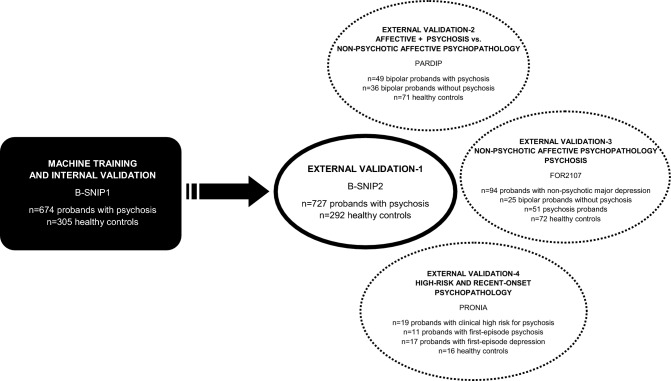
Overview of study samples that were included into the machine training and validation procedures.

To provide comprehensive internal and external validation in the present study, we developed a machine-learning based model that was trained on a set of traditional measures characterizing specific SPEM subfunctions, i.e. mean eye velocity, initial eye acceleration and initiation latency^20^, in the large sample of the B-SNIP1 study. We then applied several external validation steps to determine stability and specificity in an independent sample of psychosis probands (external validation-1: B-SNIP2 sample), in bipolar probands with and without psychosis symptoms (external validation-2: Psychosis and Affective Research Domains and Intermediate Phenotypes (PARDIP) sample), in probands with predominately affective disorders as well as psychosis probands (external validation-3: DFG-Forschergruppe 2107 (FOR2107) sample) and, following an exploratory approach, in clinical high risk as well as recent-onset psychosis and depression states (external validation-4: Personalised Prognostic Tools for Early Psychosis Management (PRONIA) sample), see Fig. 1. Our aim was to develop an algorithm based on SPEM characteristics which allows evaluation of psychosis-related sensorimotor transformation function on an individual level. Ideally, such SPEM characteristics can be assessed in a short 5-min test ensuring practical utility.

## Results

Demographics, clinical characteristics, and SPEM descriptive information for proband groups by study can be found in Tables 1, 2. With regard to the B-SNIP1 sample, there were no significant differences for age (T(977) = 1.20, p = 0.23) and sex (χ(1) = 1.16, p = 0.28) between psychosis probands and healthy controls. However, healthy controls yielded higher cognition scores than psychosis probands (T(953) = 5.93, p < 0.001). Correlations between SPEM performance and possible confounds, i.e. cognition scores or chlorpromazine equivalents were negligible, see Supplementary Tables 8–10.

**Table 1:** Descriptive information and clinical characteristics for proband groups by study.

Study	N	Age	Sex	Cognition^c^	Illness duration	Psychosis^d^		Depression^e^	Mania^f^	Medication^g^
			% Male	Total score	(Years)	Positive	Negative	Total score	Total score	CPZ
B-SNIP1 (machine training and internal validation)^h^										
Controls	305	36.5 (12.4)	45	103.80 (14.00)						
Psychosis probands^a^	674	35.5 (12.5)	49	97.66 (15.19)	15.41 (11.91)	15.89 (5.64)	14.81 (5.43)	10.74 (9.32)	5.91 (6.29)	467.11 (438.99)
SZ	265	34.5 (12.5)	67	94.88 (16.02)	13.58 (11.77)	17.07 (5.68)	16.56 (5.64)	8.72 (8.30)	5.63 (5.83)	522.83 (435.50)
SAD	178	36.3 (11.6)	40	96.68 (14.90)	16.64 (11.24)	18.04 (5.36)	16.03 (5.21)	14.29 (9.68)	7.28 (6.62)	531.03 (526.92)
BP	231	36.0 (13.00)	35	101.59 (13.59)	16.55 (12.34)	12.92 (4.47)	11.90 (3.96)	10.25 (9.38)	5.20 (6.42)	340.09 (317.84)
B-SNIP2 (external validation-1)^i^										
Controls	292	33.4 (11.2)	40	101.13 (11.17)						
Psychosis probands^a^	727	38.6 (12.2)	50	93.53 (14.30)	17.92 (12.89)	16.16 (6.78)	14.90 (7.00)	11.41 (10.24)	8.11 (7.78)	538.87 (1001.07)
SZ	288	39.8 (12.5)	60	90.67 (13.76)	18.94 (12.73)	17.18 (6.75)	17.04 (7.10)	9.15 (9.10)	7.63 (6.65)	653.12 (1137.71)
SAD	264	40.1 (11.8)	45	91.78 (13.69)	21.05 (13.21)	17.51 (6.92)	14.84 (6.85)	13.27 (10.48)	9.64 (8.24)	539.97 (1064.84)
BP	175	34.1 (11.0)	41	101.90 (13.24)	11.32 (10.01)	12.48 (5.15)	11.56 (5.64)	12.15 (10.94)	6.47 (8.28)	297.47 (278.29)
PARDIP (external validation-2)^j^										
Controls	71	37.7 (13.4)	55	103.65 (15.16)						
BPwP	49	41.1 (11.2)	43	92.46 (15.46)	11.9 (8.7)	16.22 (6.60)	16.84 (6.84)	17.86 (12.46)	12.14 (9.20)	339.61 (328.08)
BPwoP	36	39.4 (13.1)	25	93.49 (13.81)	9.4 (9.6)	13.76 (3.74)	17.41 (6.98)	17.15 (11.05)	10.15 (7.86)	164.43 (122.31)
FOR2107 (external validation-3)^k^										
Controls	72	34.2 (13.2)	39	112.42 (14.92)		11.22 (0.14)	7.34 (0.56)	2.71 (3.51)	0.53 (1.17)	
Psychosis probands^b^	51	35.0 (10.2)	69	109.67 (11.19)	9.6 (9.6)	13.43 (3.33)	13.21 (5.99)	13.89 (11.19)	1.67 (1.41)	675.75 (370.33)
MDwoP	94	35.0 (11.7)	40	112.78 (13.41)	6.4 (7.3)	11.34 (0.36)	10.89 (4.32)	12.16 (9.60)	1.09 (1.95)	98.52 (139.15)
BPwoP	25	38.4 (11.6)	48	113.69 (19.32)	8.5 (8.9)	11.47 (0.75)	10.91 (4.18)	11.88 (13.45)	1.69 (1.97)	244.34 (151.94)
PRONIA (external validation-4)^l^										
Controls	16	24.3 (3.6)	31	11.85 (2.15)		7.06 (0.25)	7.00 (0.00)	3.07 (2.56)		
ROD	17	20.7 (5.5)	59	9.43 (1.81)	0.7 (0.5)	7.00 (0.00)	14.88 (6.23)	22.71 (11.36)		31.67 (17.62)
CHR	19	21.0 (3.6)	37	11.57 (1.70)		9.44 (2.13)	14.13 (4.92)	31.06 (8.98)		80.95 (67.60)
ROP	11	24.1 (6.5)	36	11.63 (1.77)	0.6 (0.6)	19.91 (8.11)	19.27 (9.80)	28.10 (16.39)		152.15 (101.87)

*CPZ equivalents* Chlorpromazine equivalents, *B-SNIP* Bipolar-Schizophrenia Network on Intermediate Phenotypes, *SZ* probands with schizophrenia, *SAD* probands with schizoaffective disorder, *BP* probands with bipolar disorder, *PARDIP* Psychosis and Affective Research Domains and Intermediate Phenotypes, *BPwP* bipolar probands with psychosis, *BPwoP* bipolar probands without psychosis, *FOR2107* DFG Forschergruppe 2107, *MDwoP* probands with major depression without psychosis, *PRONIA* Personalised Prognostic Tools for Early Psychosis Management, *ROD* recent-onset depression probands, *CHR* clinical-high-risk- for psychosis probands, *ROP* recent-onset-psychosis probands.

Table represents means and standard deviations given in parentheses. ^a^Group of psychosis probands include SZ, SAD, and BP probands, ^b^Group of psychosis probands include n = 27 SZ, n = 18 SAD, n = 2 Brief psychotic disorder, n = 1 Delusional disorder, n = 2 BPwP, n = 1 MDwP; due to small samples, no specific results for psychosis subgroups are given, ^c^Total score indicating cognition abilities were estimated using the following measures: B-SNIP1, B-SNIP2, PARDIP = Wide Range Achievement Test 4 (WRAT4 ^55^; FOR2107 = Multiple Choice Vocabulary Test, version B (MWT-B ^74^, original MWT-B scores were transformed to the IQ scale ^74^; PRONIA = Wechsler Adult Intelligence Scale Matrix Reasoning ^75^. ^d^Psychosis features were estimated using the following measures the following measures: B-SNIP1, B-SNIP2, PARDIP, and PRONIA = Positive And Negative Syndrome Scale (PANSS ^66^ ); FOR2107 = Scale for Assessment of Positive Symptoms (SAPS) and Scale for Assessment of Negative symptoms (SANS) ^67^, SAPS and SANS global/summary scores were converted to PANSS scores ^68^; ^e^Depressive symptoms were estimated using the following measures: B-SNIP1, B-SNIP2, PARDIP = Montgomery-Åsberg Depression Rating Scale (MADRS ^69^); FOR2107 = Original Beck Depression Inventory, 1978 version ^70^; PRONIA = Beck Depression Inventory-II (BDI-II ^71^); ^f^For B-SNIP1, B-SNIP2, PARDIP and FOR2107 sample, mania was estimated using the Young Mania Rating scale ^73^. Mania was not assessed in the PRONIA sample. ^g^Chlorpromazine equivalents were assessed based on the computations by Andreasen and colleagues ^80^. ^h^Significant differences between controls and psychosis probands were found for cognition total score (p < .001). ^i^Significant differences between controls and psychosis probands were found for age (p < .001), sex distribution (p = .003) and cognition total score (p < .001). ^j^Significant differences were found for sex distribution (p = 0.01), cognition total score (p < 0.001; controls > BPwP, controls > BPwoP), and psychosis positive (p = 0.03). ^k^Significant differences were found for sex distribution (p = .004), psychosis positive (p < .001; psychosis probands > controls and MDwoP and BPwoP), psychosis negative (p < 0.001; psychosis probands > controls and MDwoP, controls < psychosis probands and MDwoP and BPwoP), depression (p < .001; psychosis probands and MDwoP and BPwoP > controls), mania (p = .04; post-hoc tests Bonferroni-corrected were all non-significant), and medication (p > 0.001; psychosis probands > BPwoP and MDwoP). ^l^Significant differences were found for psychosis positive (p < 0.001; ROP > ROD and CHR and controls), psychosis negative (p < 0.001; ROP and ROD and CHR > controls), and depression (p < 0.001; ROP and ROD and CHR > controls).

**Table 2:** Descriptive results of smooth pursuit eye movements for proband groups by study.

Study	N	Maintenance gain (%/100)		Early gain (%/100)		Initial eye acceleration (°/sec^2^)		Latency (ms)	
		Mean	SD	Mean	SD	Mean	SD	Mean	SD
B-SNIP1 (machine training and internal validation)^a^									
Controls	305	0.93	0.10	0.79	0.19	80.28	35.49	175.84	26.59
Psychosis probands	674	0.86	0.17	0.64	0.25	61.54	34.52	183.75	37.23
SZ	265	0.84	0.19	0.62	0.26	60.30	37.23	182.01	40.01
SAD	178	0.88	0.15	0.66	0.24	63.13	34.00	188.90	38.00
BP	231	0.86	0.17	0.67	0.24	61.76	31.66	181.80	32.88
B-SNIP2 (external validation-1)^b^									
Controls	292	0.93	0.05	0.76	0.15	80.99	30.62	180.01	30.61
Psychosis probands	727	0.85	0.13	0.59	0.21	72.91	37.84	196.18	43.33
SZ	288	0.85	0.13	0.57	0.21	70.16	38.85	195.88	45.70
SAD	264	0.84	0.14	0.57	0.21	68.68	37.00	198.29	43.07
BP	175	0.88	0.12	0.65	0.19	83.81	35.46	193.53	39.62
PARDIP (external validation-2)^c^									
Controls	71	0.91	0.11	0.76	0.18	75.85	31.93	184.94	32.66
BPwP	49	0.81	0.16	0.58	0.22	57.32	30.82	191.13	49.20
BPwoP	36	0.87	0.14	0.70	0.19	65.52	29.23	188.25	43.69
FOR2107 (external validation-3)^d^									
Controls	72	0.90	0.08	0.72	0.17	97.87	32.79	164.46	15.94
Psychosis probands	51	0.85	0.15	0.61	0.18	83.07	29.07	164.53	16.73
MDwoP	94	0.90	0.09	0.72	0.15	103.92	34.00	162.56	14.65
BPwoP	25	0.87	0.10	0.65	0.15	82.08	28.40	168.91	15.51
PRONIA (external validation-4)^e^									
Controls	16	0.91	0.06	0.77	0.10	107.79	24.08	165.36	18.66
ROD	17	0.89	0.10	0.70	0.19	114.32	42.53	169.19	21.96
CHR	19	0.81	0.22	0.60	0.22	99.46	34.92	178.34	17.49
ROP	11	0.90	0.08	0.67	0.14	114.23	46.02	168.59	17.22

*B-SNIP* bipolar-schizophrenia network on intermediate phenotypes, *SZ* probands with schizophrenia, *SAD* probands with schizoaffective disorder, *BP* probands with bipolar disorder, HC healthy controls, *PARDIP* psychosis and affective research domains and intermediate phenotypes, BPwP bipolar probands with psychosis, *BPwoP* bipolar probands without psychosis, *FOR2107* DFG Forschergruppe 2107, *MDwoP* probands with major depression without psychosis, PRONIA personalised prognostic tools for early psychosis management, *ROD* recent-onset depression probands, *CHR* clinical-high-risk- for psychosis probands, *ROP* recent-onset-psychosis probands, *SD* standard deviation.

^a^Significant differences between controls and psychosis probands were found for maintenance gain, early gain, initial eye acceleration, and latency (all p > .001). ^b^Significant differences between controls and psychosis probands were found for maintenance gain, early gain, initial eye acceleration, and latency (all p > 0.001). ^c^Significant differences were found for maintenance gain (p < 0.001; BPwP < controls), early gain (p < 0.001; BPwP < controls and BPwoP), and initial eye acceleration (p = 0.008; BPwP < controls). ^d^Significant differences were found for maintenance gain (p = 0.009; psychosis probands < controls and MDwoP), early gain (p < 0.001; psychosis probands < controls and MDwoP), and initial eye acceleration (p < 0.001; psychosis probands < MDwoP, BPwoP < MDwoP). ^e^Significant differences were found for early gain (p < 0.001; CHR < controls).

### Machine training and internal validation: B-SNIP1

The model distinguished psychosis probands from healthy controls by SPEM variables with a mean balanced accuracy of 63.96% (p < 0.001, Table 3; for further results parameter refer to Supplementary Table 4). On average 53% of the psychosis probands and 75% of the control subjects were correctly classified (sensitivity = 52.97%, specificity = 74.96%, Table 3). Mean likelihood ratios^42^ resulted in: positive test result = 2.18, negative test result = 0.63.

**Table 3:** Prediction accuracies for all samples and model results for the comparison of chronic psychosis probands vs. controls.

	True label	Predicted label		BAC^b^	Sensitivity^b^	Specificity^b^
		Psychosis probands n (%)	Controls n (%)			
B-SNIP1 (training and internal validation)						
Psychosis probands vs. controls, fold 1	Controls (n = 104)	32 (30.77%)	72 (69.23%)	61.97	54.71	69.23
	Psychosis probands (n = 223)	122 (54.71%)	101 (45.29%)			
Psychosis probands vs. controls, fold 2	Controls (n = 89)	22 (24.72%)	67 (75.28%)	63.80	52.32	75.28
	Psychosis probands (n = 237)	124 (52.32%)	113 (47.68%)			
Psychosis probands vs. controls, fold 3	Controls (n = 112)	22 (19.64%)	90 (80.36%)	66.11	51.87	80.36
	Psychosis probands (n = 214)	111 (51.87%)	103 (48.13%)			
Mean				**63.96**^a^	**52.97**	**74.96**
B-SNIP2 (external validation-1)						
Psychosis Probands vs. controls	Controls (n = 289)	75 (25.95%)	214 (74.05%)	**65.03**	**56.01**	**74.05**
	Psychosis probands (666)	373 (56.01%)	293 (43.99%)			
PARDIP (external validation-2)						
BPwP vs. controls				**65.52**	**68.18**	**62.86**
	Controls (n = 70)	26 (37.14%)	44 (62.86%)			
	BPwP (n = 44)	30 (68.18%)	14 (31.82%)			
	BPwoP (n = 33)	13 (39.39%)	20 (60.61%)			
FOR2107 (external validation-3)						
Psychosis probands vs. controls				**58.37**	**43.14**	**73.61**
	Controls (n = 72)	19 (26.39%)	53 (73.61%)			
	Psychosis Probands (n = 51)	22 (43.14%)	29 (56.86%)			
	MDwoP (n = 94)	18 (19.15%)	76 (80.85%)			
	BPwoP (n = 25)	10 (40.00%)	15 (60.00%)			
PRONIA (external validation-4)	Controls (n = 16)	1 (6.25%)	15 (93.75%)			
	ROD (n = 17)	4 (23.53%)	13 (76.47%)			
	CHR (n = 19)	8 (42.11%)	11 (57.89%)			
	ROP (n = 11)	2 (18.18%)	9 (81.82%)			

*B-SNIP* bipolar-schizophrenia network on intermediate phenotypes, *PARDIP* psychosis and affective research domains and intermediate phenotypes, *BPwP* bipolar probands with psychosis, *BPwoP* bipolar probands without psychosis, FOR2107 DFG Forschergruppe 2107, *MDwoP* probands with major depression without psychosis, *PRONIA* personalised prognostic tools for early psychosis management, *ROD* recent-onset depression probands, *CHR* clinical-high-risk- for psychosis probands, *ROP* recent-onset-psychosis probands, *BAC* balanced accuracy score.

^a^p-value < 0.001, indicated for BAC score as this metric was used to identify the best performing model. ^b^BAC, sensitivity, and specificity can only be computed for comparisons including psychosis patients and controls (as these groups from the B-SNIP1 sample were used in the machine training and internal validation processes). For all other samples true label and predicted label (psychosis proband or healthy control) are given.

Main results are displayed in bold.

### External validation-1: B-SNIP2

Validation in the B-SNIP2 sample included n = 666 psychosis probands and n = 289 healthy controls (n = 64 participants could not be entered into the machine due to at least one missing value). Emphasizing high validity, the B-SNIP1 derived model discriminated psychosis probands from healthy controls in the independent B-SNIP2 sample with a balanced accuracy of 65.03% (see Table 3 and Supplementary Table 4). About 56% of the psychosis probands and 74% of the control subjects were correctly classified (sensitivity = 56.01%, specificity = 74.05%, Table 3).

### External validation-2: PARDIP

For the PARDIP sample, n = 44 bipolar probands with psychosis symptoms, n = 33 bipolar probands without psychosis symptoms and n = 70 healthy controls were included in the validation procedure (n = 9 participants were excluded due to at least one missing value). Our trained model could distinguish bipolar probands with psychosis symptoms from healthy controls with a balanced accuracy of 65.52% (Table 3 and Supplementary Table 4). About 68% of the bipolar probands with psychosis were correctly classified as psychosis probands and 63% of the control subjects were correctly classified as healthy controls (sensitivity = 68.18%, specificity = 62.86%, Table 3). Furthermore, about 61% of the bipolar probands without psychosis symptoms were classified as controls (which means that they are closer to the healthy non-psychotic than the psychosis category, Table 3).

### External validation-3: FOR2107

To validate the machine in predominately affective psychopathology, data from n = 94 probands with major depression and n = 25 probands with bipolar disorder, both groups without psychotic symptoms, from the FOR2107 consortium were entered into the analyses. Using the B-SNIP1 machine, nearly 81% of the probands with major depression and 60% of the probands with bipolar disorder were classified as being closer to the healthy non-psychotic than the psychosis category, Table 3.

As proof of principle, we also validated the B-SNIP1 machine on n = 51 psychosis probands and n = 72 healthy controls from FOR2107 revealing a balanced accuracy of 58.37% (Table 3 and Supplementary Table 4). In detail, about 43% of the psychosis probands and 74% of the control subjects were correctly classified (sensitivity = 43.14%, specificity = 73.61%, Table 3).

### External validation-4: PRONIA

Validation in high-risk and recent-onset psychotic or depressive disorder could be computed in n = 11 probands with recent-onset psychosis, n = 17 probands with recent-onset depression, n = 19 participants with clinical high risk of psychosis, and n = 16 controls (PRONIA study). Emphasizing the validity of the machine, about 94% of the controls were categorized as healthy. However, in contrast to previous results in chronically ill psychosis probands, only 18% of the recent-onset psychosis probands were classified as psychosis patients (Table 3). Interestingly, the machine labeled nearly 42% of the participants with clinical high risk of psychosis as psychosis probands (Table 3). Of the probands with recent-onset, non-psychotic depression, 76% were classified as healthy controls (Table 3).

### Effects of sample size on model performance

Training models in reduced (randomly selected 50% of the B-SNIP1 sample) and larger (combined B-SNIP1 and B-SNIP2) samples showed that balanced accuracies (50% B-SNIP1 = 62.28%, B-SNIP1 = 63.96%, B-SNIP1 + B-SNIP2 = 65.87%), specificities (50% B-SNIP1 = 78.48%, B-SNIP1 = 74.96%, B-SNIP1 + B-SNIP2 = 80.94%) and sensitivities (50% B-SNIP1 = 46.08%, B-SNIP1 = 52.97%, B-SNIP1 + B-SNIP2 = 50.80%) were rather unaffected by sample size (Supplementary Tables 6, 7 and Supplementary Fig. 1).

## Discussion

In the current study we examined a set of traditional SPEM measures (i.e. predictive eye velocity maintenance gain, early eye velocity maintenance gain, initial eye acceleration, and eye latency; Leigh & Zee^17^; Lencer et al.^20^) and their interactions as quantifiable biological indicators of psychosis-related visual sensorimotor dysfunction in large samples of probands with psychotic disorders. This is an important approach since identified SPEM deteriorations point to specific deficits in the transformation of sensory motion signals into motor action being associated with alterations in occipito-parieto-frontal networks^24,43^.

To overcome limitations by classical frequentist statistics, we implemented multivariate pattern analyses (e.g. supervised machine learning approaches)^44^ using internal (i.e. a hold-out subsample consisting of participants that were not used for training) and external (i.e. an independent dataset) validation in sufficient large data samples^11^ to allow for clinically relevant single-subject statements pointing to sensorimotor transformation deficits. Most importantly, we not only trained and internally validated the machine-learning algorithm in a single sample but also applied and externally validated the machine in an independent large sample of psychosis probands and healthy controls (external validation-1: B-SNIP2), in a sample of bipolar probands with and without psychotic symptoms (external validation-2: PARDIP), in a sample of probands with affective disorders without psychotic symptoms and psychosis probands (external validation-3: FOR2107), and in a sample with recent-onset psychosis or depression and clinical high risk of psychosis (external validation-4: PRONIA). Our main finding shows high consistency for the identification of psychosis probands vs. healthy controls by these sensorimotor indicators throughout the four different samples (B-SNIP1: 63.96%, B-SNIP2: 65.03%, PARDIP: 65.52%, FOR2107: 58.37%). However, it is important to consider that our model performed notably better in accurately classifying controls as controls (specificities in the different samples ranged from 63 to 75%) than psychosis probands as psychosis probands (sensitivities ranged from 43 to 68%).

Although a balanced accuracy score of nearly 64% as derived from our training sample (B-SNIP 1) may be regarded as insufficient for SPEM performance to be used as a single screening instrument for determining psychosis-related sensorimotor transformation function, it significantly exceeds chance level and remains within the range of expectable results in similar heterogenous psychiatric sample sizes^11^. Additionally, a likelihood ratio for a positive test result of 2.18 could be interpreted as small (but important) changes in probability^42^. Our second key finding emphasizes the generalization to new data when applying the model to an independent cohort of chronically ill psychosis probands and healthy controls. Regarding the first external validation in the B-SNIP2 sample (external validation-1), our machine yielded a comparable (even slightly higher) balanced accuracy of 65.03% when discriminating the two groups. This result is particularly meaningful due to (a) the independence of both data sets and (b) slight differences in the SPEM task design underlining the robustness of classification results by our model. A third cohort with chronically ill psychosis probands and healthy controls was derived from the FOR2107 consortium (external validation-3) and could be classified correctly with a balanced accuracy of 57.64%.

Our findings support the original suggestions by Diefendorf and Dodge^45^ to use SPEM as a neurobiological diagnostic tool coming with multiple advantages including standardized measurements and brief 5-min testing feasible even for severely impaired patients. Here, we applied a constellation of SPEM tasks consisting of full-ramp and foveo-petal step-ramp trials at 18.7 degrees of visual angle constant velocity. These specific SPEM tasks allow the computation of the four key measures to evaluate SPEM performance and can be recommended for future studies. Our results add to previous findings based on traditional group analyses in indicating that SPEM is a valuable psychosis-related biomarker of sensorimotor integrity being useful even at the single-subject level^20^. Besides its diagnostic value this biomarker bears highly relevant information for establishing personalized treatment regimes.

Very recently St Clair and colleagues^46^ applied a multiclass machine-learning model to differentiate patients with schizophrenia, bipolar affective disorder, major depression disorder, and healthy controls on the basis of 98 eye movement symptoms (including several SPEM variables). The model was tested in two validation sets achieving balanced accuracies for schizophrenia patients of 73% and 75%. Both validation sets were relatively small (test-1 internal validation: n = 30 schizophrenia, n = 35 bipolar, n = 33 depression, n = 35 controls; test-2 external validation: n = 60 schizophrenia, n = 184 controls) which entails an increased risk of misclassification^11^. To avoid this common short coming we have used a large internal validation sample as well as applied our machine to several extensive independent data sets. Of note, the task from St Clair and colleagues took about 15 min in total yielding a total of 98 eye movement measures^47^ derived from free viewing, fixation duration, and smooth pursuit tasks^46^ limiting its clinical practicability.

To further determine the model’s specificity regarding the relationship between psychotic symptoms and SPEM performance we applied the machine to other patient groups. To this regard, there has been an extensive discussion concerning similarities and differences between schizophrenia and bipolar disorder^48^. Machine-learning models based on brain data have been used to discriminate both patient groups^49^, though often merging data from bipolar patients with and without history of psychotic episodes^50^.

Similarly, St Clair and colleagues^46^ did not specify psychosis symptoms in those patients suffering from bipolar disorder and major depression which we found has a significant impact as demonstrated by our external validation-2 sample from the PARDIP. In line with the idea of the relationship between SPEM deterioration and psychotic psychopathology, our machine classified about 68% of the bipolar probands with psychosis correctly as psychosis patients, while 61% of the bipolar probands without psychosis symptoms were classified as healthy (which means that they are closer to healthy individuals). Underlining its generalizability, 60% of the bipolar probands without psychotic symptoms from the FOR2107 study (external validation-3) were also rated closer to the healthy non-psychotic category.

Broadening the perspective of specificity regarding SPEM deficits in affective disorders, we found that nearly 81% of probands suffering from major depression without psychotic episodes (FOR2107 study, external validation-3) were classified as healthy indicating closer affiliation to the non-psychotic category. This result is in line with previous findings of only minor impaired SPEM performance from traditional group statistics^36^ and multivariate pattern analyses based on brain data indicating major depression and schizophrenia as two end points of an interjacent continuum^50^.

Our external-validation sample 4 from the PRONIA study was used to test our model in young probands being at clinical high risk for psychosis or experiencing a first psychotic or first depressive episode. Interestingly, about 42% of probands with clinical high risk of psychosis were categorized as psychosis probands which might support the idea of an underlying susceptibility of SPEM deficits in the psychosis spectrum^51^. Indeed, the specific SPEM measures of predictive and early maintenance gain indicated the worst performance in this proband group compared to all three other PRONIA groups (see Table 2). However, this group is extremely heterogeneous as indicated by large standard deviations in the early and maintenance gains (see Table 2). Note, transition rates for CHR to ROP are about 25% within 3 years indicating a high heterogeneity of CHR subjects regarding susceptibility to psychosis^52^. In contrast, in the relatively small (n = 11) and heterogeneous sample of recent-onset psychosis probands our machine only classified two probands (18%) as belonging to the psychosis group. Despite the small sample size, this observation points to possible differences in SPEM performance between recent-onset and chronic states of psychosis (see also Table 1 for information about illness duration) as discussed previously^53^. That study observed subtle impairments of immediate sensorimotor processing in first-episode psychosis patients with only short duration of treatment, e.g. after 6 weeks, which appeared to be compensated by predictive drive to pursuit. In more detail, first-episode patients demonstrated slightly worse performance in the pure-ramp task (comparable to the step-ramp task in the current study) but were unaffected in the oscillating task (comparable to the triangle wave task in the current study). Deficits were discussed as possible medication effects with regard to their serotonergic antagonism of brainstem sensorimotor systems. However, same as in the present study, no associations between SPEM variables and medication dosage were found^53^. Indeed, in our ROP group (which might be comparable to the first-episode patients after short duration of treatment from the study by Lencer and colleagues^53^), early maintenance gain -driven by immediate sensorimotor processing- was considerably reduced while predictive maintenance gain was unaffected (see Table 2). Notably, 76% of probands with recent-onset depression and 94% of healthy controls from the PRONIA sample were correctly classified as not belonging to the psychosis group.

Despite the clear strengths of the study, some limitations need to be discussed: (1) SPEM results for initial eye acceleration and latency differed between laboratories/recording devices (Supplementary Table 11). To estimate the impact of these two variables on the prediction of our machine, we additionally trained a machine in the B-SNIP1 sample using only the two eye velocity gain measures as predictors. The machine was able to distinguish psychosis probands from healthy controls with a balanced accuracy of 61.90% (Supplementary Table 12) which is close to the main result using all SPEM variables (63.96%). However, laboratory conditions and/or recording devices may have an impact on the measurement of SPEM initial eye acceleration and latency that could have affected prediction results. (2) As we trained the machine in a sample of chronically ill psychosis probands, possible effects of medication have to be taken into account. Although we found only small and inconsistent correlations between SPEM and chlorpromazine equivalents, effects of medication cannot be fully ruled out^53^. (3) Furthermore, we found significant differences in cognition scores between psychosis probands and healthy controls in the B-SNIP1 sample. There might be effects of cognitive skills that cannot be entirely discarded. (4) Despite our comprehensive validation samples, our machine was not validated in a group of MDD with psychosis. (5) There is a discrepancy between sensitivity (53%) and specificity (75%) implying our model to be particularly suitable to correctly identify healthy probands as healthy. (6) No follow-up data of samples from the PRONIA study is available to evaluate transition rates of those CHR participants with bad SPEM performance.

Our comprehensive findings support SPEM as an indicator of sensorimotor transformation impairments relevant to patients suffering from chronic psychosis. Thus, our machine learning algorithm based on the performance in a 5 min SPEM task can help to obtain an overview of sensorimotor transformation profiles on an individual level that might inform treatment decisions in rehabilitation contexts, e.g. regarding sensorimotor remediation strategies.

Future studies should broaden this biomarker approach by combining indicators of sensorimotor function with multiple other relevant neurobiological measures, e.g. brain structure indices, to improve individual prediction accuracies and to inform personalized therapeutic decisions for psychotic disorders. Additionally, future studies should target the question whether SPEM-Impairments can indicate illness progression independently from the factor of illness duration.

## Methods

### Subjects

SPEM data from five independent samples were included in the following analyses (Fig. 1):

#### B-SNIP1

First, the machine was trained and internally validated with SPEM data from the B-SNIP1 sample consisting of n = 674 chronically ill psychosis probands (n = 265 schizophrenia, n = 178 schizoaffective, and 231 bipolar with psychotic symptoms) and 305 healthy controls. Participants were recruited by the B-SNIP consortium across five sites in the US (Baltimore, Boston, Chicago, Dallas, Hartford; Tamminga et al.^27^). Diagnoses were derived by a consensus of experienced clinicians based on all available clinical information and the Structured Clinical Interview for DSM-IV^54^. Inclusion criteria comprised (1) age between 15 and 65 years; (2) reading score of the Wide Range Achievement Test ≥ 60^55^; (3) no history of a neurologic disorder; (4) normal or corrected to normal vision (minimum of 20/40 acuity), (5) no history of substance abuse within the last month or substance dependence within the last three months, and negative urine toxicology on study day. Additionally, healthy controls were not allowed to have a personal or family history (first-degree) of psychotic or bipolar disorders, to have a history of recurrent mood disorder or to exhibit a history of psychosis spectrum personality traits^56^. The protocol of the study was approved by institutional review boards at each of the study sites and participants provided written informed consent. For group differences in SPEM performance see Lencer et al.^20^.

Second, the remaining study samples were used (a) as external validation data for the machine trained in the B-SNIP1 sample and (b) for investigating psychosis-related specificity of SPEM against probands with predominately affective disorders.

#### External validation-1: B-SNIP2

B-SNIP2 is the follow-up to B-SNIP1. SPEM data were available from n = 727 chronically ill psychosis probands (n = 288 schizophrenia, n = 264 schizoaffective, and 175 bipolar with psychotic symptoms) as well as n = 292 healthy controls recruited in Boston, Chicago, Dallas, Hartford, and Athens (GA). Inclusion criteria were identical to B-SNIP1. For further details on B-SNIP2 eye movements see Huang et al., 2021^62^, but SPEM data have not been published so far.

#### External validation-2: PARDIP

SPEM data from the multisite PARDIP consortium were available from n = 49 bipolar probands with psychotic symptoms (BPwP), n = 36 bipolar probands without psychotic symptoms (BPwoP), and n = 71 healthy controls. The PARDIP study took place in Dallas, Boston, and Hartford. It was nested within the B-SNIP consortium using similar inclusion criteria but, importantly, there was no overlap between PARDIP and B-SNIP participants. Further information on inclusion criteria and group differences in SPEM performance see Brakemeier et al.^57^.

#### External validation-3: FOR2107

In collaboration with the bicentric FOR2107 project (https://for2107.de/, Kircher et al.^58^), SPEM data were measured in n = 94 probands with major depressive disorder without psychotic symptoms (MDwoP), n = 25 bipolar probands without psychotic symptoms (BPwoP), n = 51 psychosis probands, and n = 72 healthy controls at the Münster site.

#### External validation-4: PRONIA

Following an exploratory approach for testing the validity of our machine developed in stable probands with chronic psychosis, SPEM data were also collected in collaboration with the multisite PRONIA consortium (https://www.pronia.eu/, Koutsouleris et al.^59^) from n = 11 probands with recent-onset psychosis, n = 19 probands with a high clinical risk for the development of psychosis, n = 17 probands with recent-onset depression, and n = 16 healthy controls at the Münster site.

All patients were medicated as prescribed by their doctors except for regular or current sedative medication which was an exclusion criterium (see chlorpromazine equivalents at time of testing in Table 1). Note, prior to inclusion ROP patients from the PRONIA sample had not been allowed to take any antipsychotic medication for longer than 90 days (within the past 24 months) with a daily dose rate at or above the minimum dosage of DGPPN S3-guidelines^60^.

Participants gave written informed consent according to the Declaration of Helsinki. Each study was approved by the respective local ethics committee.

### Eye movement measurement and task

At all sites, the SPEM target consisted of a small stimulus (0.5°) moving back and forth in the horizontal plane at 18.7°/s constant velocity displayed on a monitor to constitute full-ramp trials within triangle wave tasks and foveo-petal step-ramp trials^61^. Participants were instructed to follow the stimulus with their eyes as accurately as possible while sitting in front of the monitor with their heads stabilized using a chin and forehead restraint. Across all studies, eye movements were recorded in a quiet and darkened room.

For B-SNIP1, B-SNIP2, and PARDIP samples (for further details refer to Brakemeier et al.^57^; Huang et al.^62^; Lencer et al.^20^), participants were seated 60 cm from a 22-inch CRT monitor (1360 × 768 resolution; 150 Hz refresh rate) and eye movements were recorded using an Eyelink II (SR Research Ltd., Ontario/Canada) recording device at 500 Hz sampling rate. The stimulus comprised a red cross in a box covering 0.5° moving horizontally between ± 12° across the screen.

In B-SNIP1 and PARDIP studies, 48 full-ramp and 32 foveo-petal step-ramp trials^61^ both at 18.7° of visual angle per second constant velocity, were applied in order to assess SPEM performance. In full-ramp trials, the stimulus moved back and forth with constant velocity in a triangular waveform. During step-ramp trials, the target started from the central position, stepped either to the right or the left (2.4° of visual angle in a randomized order) and afterwards moved towards the peripheral opposite direction at 18.7° of visual angle per second constant velocity. The stimulus re-crossed the central line after 133 ms allowing the initiation of SPEM without a necessary catch-up saccade^61^. Additionally, some trials with 9.7° of visual angle/second and 26.6° of visual angle/second target velocities as well as trials with intervals where the target was blanked were displayed to enhance attention but were not included into the analyses (30% of trials). In order to ensure data quality, additional calibration trials were presented between blocks of trials. SPEM measurement was conducted identically across sites.

For B-SNIP2 a slightly different task order was applied: Here, 48 full-ramp trials at 18.7° of visual angle per second constant velocity and a total of 48 foveo-petal step-ramp trials (32 × 18.7° of visual angle/second; 8 × 9.7° of visual angle/second; 8 × 26.6° of visual angle/second; randomized for direction; Rashbass^61^) were presented in two test sets (each consisting of 48 trials) including six alternating blocks of either eight full-ramp or step-ramp trials. Step-ramp trials at 9.7° of visual angle/second and 26.6° of visual angle/second velocities were shown to enhance attention but were not included into the analyses. To ensure data quality, additional calibration trials were displayed between blocks of trials. SPEM measurement was conducted identically across sites.

FOR2107 and PRONIA eye movements were recorded using an Eyelink 1000 (SR Research Ltd., Ontario/Canada) recording device at 500 Hz sampling rate. Participants were seated 60 cm from a 22-inch CRT monitor (1360 × 768 resolution; 150 Hz refresh rate). Stimulus and task were identical to B-SNIP2.

### Eye movement data processing

All SPEM data were analyzed using the identical routines in MatLab (The MathWorks, Natick, MA) developed by one of the authors (AS). Eye position data were filtered using a one-dimensional Gaussian filter (30 Hz) and, subsequently, smoothed eye velocity was computed with central median differentiation of 9 ms^20,57,63^. Sections of saccades and blinks were automatically detected and excluded from computations of SPEM variables. To revise automatic calculations, individual velocity traces were checked by visual inspection.

**Figure 2:**
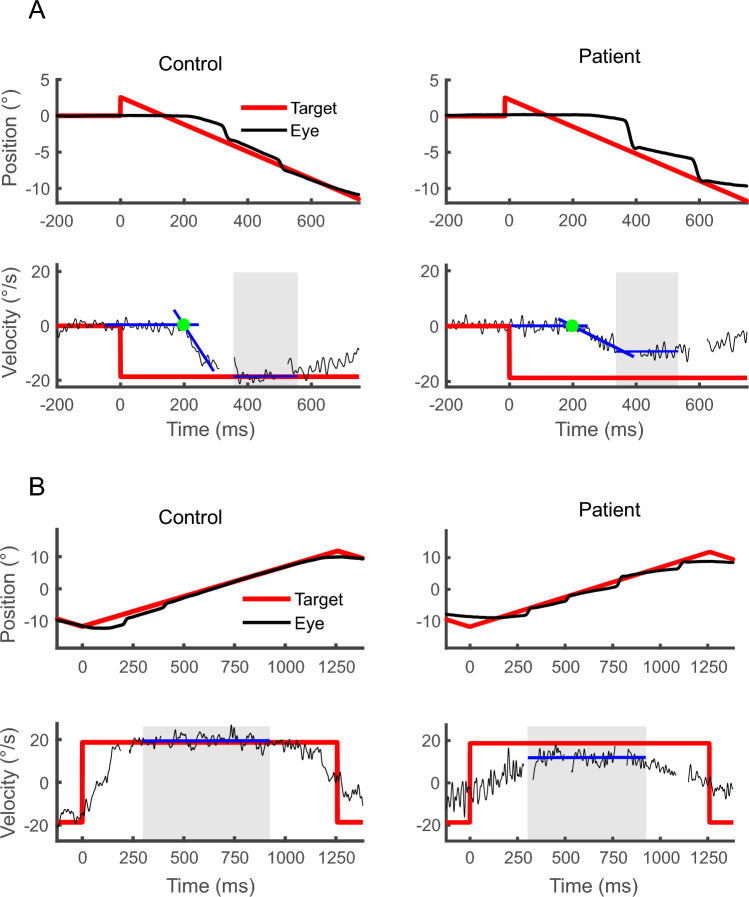
Examples of pursuit stimuli with pursuit recordings (eye position and eye velocity) in a control subject and a psychosis proband. Foveopetal step-ramp tasks (**A**) are used to measure saccade free pursuit initiation. Variables of interest are pursuit latency (time between target step and green dot), initial eye acceleration (blue line) and early maintenance gain (blue line in grey shaded intervals). Triangular wave tasks (**B**) are used to measure sustained predictive maintenance gain in predefined intervals (blue line in grey shaded intervals) excluding artifacts induced by target reversals. The figure has been adapted from one of our prior publications by Brakemeier and colleagues^57^.

To assess the different sensorimotor aspects of SPEM performance, the following variables were computed^20,36,57^ (see Fig. 2, adapted from Ref.^57^):

*Predictive maintenance gain* during continuous pursuit was calculated from triangular wave tasks as the ratio of median eye velocity to target velocity from middle sections (300–840 ms after stimulus direction reversal) over all full-ramp trials (total duration of a ramp is 1200 ms). Predictive maintenance gain highly depends on predictive drive, i.e. cognitive input to the pursuit system for sustained SPEM under closed-loop conditions.

In contrast, measures from foveo-petal step-ramp tasks represent rapid sensorimotor transformations using immediate visual motion and early performance feedback.

This included first, *early maintenance gain* as the ratio of median eye velocity to target velocity from middle sections (350-550 ms after stimulus onset) over all unpredictable step-ramp trials, thus reflecting early eye velocity under visual feedback control^64^. Typically, early maintenance gain is considerably lower compared to sustained predictive maintenance gain.

Second, for the computation of *initial eye acceleration* under open-loop conditions, when visual feedback is not yet available, eye velocity was smoothed using a Savitzky-Golay finite impulse response filter (polynomial order of 3 and a frame length of 63). The onset of eye acceleration was defined as eye velocity exceeding a noise threshold (above 3.2 standard deviations of mean resting eye velocity which was calculated from 200 ms before to 100 ms after ramp-onset, Carl & Gellman^65^) for at least 20 ms. Initial eye acceleration was then computed using robust linear regression slope (RobustFit^®^ in MatLab) in a 100 ms time window starting with the acceleration onset over all trials.

Third, *eye latency* was determined as time that had elapsed between onset of stimulus movement and onset of eye acceleration^65^ over all trials.

### Psychometric, cognitive, and clinical measures

#### Psychosis-related symptoms

For B-SNIP1, B-SNIP2, PARDIP, and PRONIA studies, psychosis-related symptoms were rated using the positive and negative syndrome scale (PANSS)^66^ while the FOR2107 study used the Scale for Assessment of Positive Symptoms (SAPS) and the scale for assessment of negative symptoms (SANS)^67^. To provide comparability, SANS and SAPS scores were converted to PANSS scores^68^, see Supplementary Table 1.

#### Depression

Depressive symptoms were quantified with the Montgomery–Åsberg Depression Rating Scale (MADRS; Montgomery & Åsberg^69^) in the B-SNIP1, B-SNIP2 and PARDIP studies and using the original Beck Depression Inventory in the 1978 version^70^ in the FOR2107 sample. For PRONIA, the Beck Depression Inventory-II (BDI-II)^71^ was applied. Severity gradation (MADRS^72^, BDI^71^) is given in Supplementary Table 2.

#### Mania

For B-SNIP1, B-SNIP2, PARDIP and FOR2107 samples, mania was estimated using the Young Mania Rating scale^73^. Mania was not assessed in the PRONIA sample.

#### Cognitive abilities

A total score indicating cognitive abilities was estimated using the Wide Range Achievement Test 4 (WRAT4^55^) in the B-SNIP1, B-SNIP2, and PARDIP samples. For the FOR2107 study, the Multiple-Choice Vocabulary Test, version B (MWT-B^74^) was used. Scores were converted to the IQ scale^74^. For the PRONIA sample the Wechsler adult intelligence scale matrix reasoning^75^ was applied to evaluate cognition.

### Statistical analyses

#### Machine learning approach

The machine learning model was trained in the B-SNIP1 sample to distinguish psychosis probands from healthy (non-psychotic) controls using PHOTONAI software^76^ and scikit-learn toolboxes^77^. A k-fold nested cross-validation procedure was applied to split data used to train the model from data taken for internal validation. Thus, to obtain the most informative model, parameters were optimized using an inner cycle (10 folds) and the best performing model chosen by highest balanced accuracy ([sensitivity + specificity]/2 taking into account imbalanced data sets) was deployed to an outer cycle (3 folds). Special attention was given to ensure that there was (1) no information leakage between train and validation data^76^ and (2) a sufficient large validation set to provide stable and meaningful results for unseen (external) samples^11^. For specifications of the best model see Supplementary Table 3.

For each of the models the following preprocessing steps were applied: (1) SPEM variables were standardized by scaling. (2) Missing values (predictive maintenance gain = 0%, early maintenance gain = 0.51%, initial eye acceleration = 1.23%, eye latency = 0.51%) were imputed with the median of the corresponding variable. (3) In order to consider different group sizes (674 psychosis probands and 305 healthy controls), data were balanced by either randomly under sampling the majority class or oversampling the minority class using SMOTE^78^. (4) Principal component analysis was applied to reduce the dimensional space.

Predictors included the four SPEM variables described above (i.e. predictive maintenance gain, early maintenance gain, initial eye acceleration, and eye latency). Then, multiple classifiers with default parameters were used to optimize representation of the underlying data (Support vector machine, Random forest, Gaussian naïve bayes, Logistic regression, Ada boost) and to discriminate the label group membership (i.e. psychosis proband or healthy control). Additionally, for the support vector machine, kernel (linear, rbf) and regularization (C = [0.1, 0.3, 0.5, 0.7, 0.9, 1]) parameters were optimized.

Statistical inference was examined using permutation tests^79^. Therefore, true results were compared to a permutation distribution created from 1000 random rearrangement of the two group labels (healthy controls vs. psychosis group) to the predictors.

Additionally, we trained machine learning algorithms to separate psychosis probands in the B-SNIP1 sample. In line with the idea of SPEM deterioration across the whole psychosis spectrum, results for distinguishing individual proband groups are close to chance level (balanced accuracies: schizophrenia vs. schizoaffective probands 52.65%, schizophrenia vs. bipolar probands 52.48%, schizoaffective vs. bipolar probands 51.00%, Supplementary Table 5).

External validation of the model was investigated by applying the best performing model from B-SNIP1 to B-SNIP2 (external validation-1), PARDIP (external validation-2), FOR2107 (external validation-3), and PRONIA (external validation-4) samples. Here, in accordance with the idea that there is a specific relationship between SPEM performance and psychosis syndromes, we also applied the model to other non-psychotic psychiatric patient groups expecting them not to be classified as psychosis probands (thus be closer to the healthy non-psychotic control group).

To examine the effect of sample size on model performance, additional models were trained and internally validated in randomly selected half of the B-SNIP1 and in the combined B-SNIP1 and B-SNIP2 samples.

Kendall’s Tau correlation coefficients were computed between SPEM measures and chlorpromazine equivalents^80^. Additionally, correlations were calculated between SPEM measures and WRAT4 scores as well as z-scores of the Brief assessment of cognition in schizophrenia (BACS; Keefe et al.^81^). Analyses were computed in the B-SNIP1 sample. Results are reported using Bonferroni–Holm-corrected alpha level adjusted for each of the studies over all four SPEM variables^82,83^.

### Supplementary Information

Supplementary Information.

## Data Availability

The data can be provided by Rebekka Lencer pending scientific review and a completed material transfer agreement. Requests for the data should be submitted to: Rebekka Lencer, lencer@uni-muenster.de.
