# Supplementary material for: Evidence from comprehensive independent validation studies for smooth pursuit dysfunction as a sensorimotor biomarker for psychosis
Source: Sci Rep. 2024 Jun 15;14:13859. doi: 10.1038/s41598-024-64487-6 (PMC11180169; doi:10.1038/s41598-024-64487-6)
Supplement: Supplementary file 1 — Supplementary Information. [file 41598_2024_64487_MOESM1_ESM.docx]

**Supplementary Material - Evidence from comprehensive independent validation studies for smooth pursuit dysfunction as a sensorimotor biomarker for psychosis**

Inga Meyhoefer^1,2,3^, Andreas Sprenger^4^, David Derad^4^, Dominik Grotegerd^1^, Ramona Leenings^1^, Elisabeth J. Leehr^1^, Fabian Breuer^1^, Marian Surmann^1^, Karen Rolfes^1^, Volker Arolt^1,2^, Georg Romer^5^, Markus Lappe^2,6^, Johanna Rehder^6^, Nikolaos Koutsouleris^7,8,9^, Stefan Borgwardt^10,11^, Frauke Schultze-Lutter^3,12,13^, Eva Meisenzahl^3^, Tilo T. J. Kircher^14^, Sarah S. Keedy^15^, Jeffrey R. Bishop^16^, Elena I. Ivleva^17^, Jennifer E. McDowell^18^, James L. Reilly^19^, Scot Kristian Hill^20^, Godfrey D. Pearlson^21^, Carol A. Tamminga^17^, Matcheri S. Keshavan^22^, Elliot S. Gershon^15^, Brett A. Clementz^18^, John A. Sweeney^23,24^, Tim Hahn^1^, Udo Dannlowski^1^, and Rebekka Lencer^1,2,10,*^

^1^ Institute for Translational Psychiatry, University of Muenster, Germany

^2^ Otto-Creutzfeldt Center for Cognitive and Behavioral Neuroscience, University of Muenster, Germany

^3^ Department of Psychiatry and Psychotherapy, Medical Faculty, Heinrich-Heine University,

Duesseldorf/LVR, Germany

^4^ Department of Neurology, University of Luebeck, Germany

^5^ Department of Child Adolescence Psychiatry and Psychotherapy, University of Muenster,

Germany

^6^ Institute of Psychology, University of Muenster, Germany

^7^ Department of Psychiatry and Psychotherapy, Ludwig-Maximilian University Munich,

Germany

^8^ Institute of Psychiatry, Psychology and Neuroscience, King’s College London

^9^ Max-Planck-Institute of Psychiatry Munich, Germany

^10^ Department of Psychiatry and Psychotherapy, University of Luebeck, Germany

^11^ Department of Psychiatry, Psychiatric University Hospital, University of Basel, Switzerland

^12^ Department of Psychology, Faculty of Psychology, Airlangga University, Surabaya,

Indonesia

^13^ University Hospital of Child and Adolescent Psychiatry and Psychotherapy, University of

Bern, Switzerland

^14^ Department of Psychiatry and Psychotherapy, Philipps-University Marburg, Germany

^15^ Department of Psychiatry and Behavioral Neuroscience, University of Chicago, USA

^16^ Department of Experimental and Clinical Pharmacology and Department of Psychiatry and

Behavioral Sciences, University of Minnesota, Minneapolis, USA

^17^ Department of Psychiatry, The University of Texas Southwestern Medical Center, Dallas, TX, USA

^18^ Departments of Psychology and Neuroscience, Bio-Imaging Research Center, University of Georgia,

Athens, GA, USA

^19^ Department of Psychiatry and Behavioral Sciences, Northwestern University Feinberg School of Medicine,

Chicago, IL, USA

^20^ Department of Psychology, Rosalind Franklin University of Medicine and Science, Chicago, IL, USA

^21^ Departments of Psychiatry and Neuroscience, Yale School of Medicine, and Olin Research Center,

Institute of Living/Hartford Hospital, Hartford, CT, USA

^22^ Department of Psychiatry, Harvard Medical School, Beth Israel Deaconess Medical Center, Boston,

MA, USA

^23^ Huaxi MR Research Center (HMRRC), Department of Radiology, West China Hospital of

Sichuan University, Chengdu, China

^24^ Department of Psychiatry and Behavioral Neuroscience, University of Cincinnati College of

Medicine, Cincinnati, USA

*Rebekka Lencer, MD

Institute for Translational Psychiatry, University of Muenster

Albert Schweitzer Campus 1, Build. A9a

48149 Muenster

Phone: +49 172 429 2225, Email: lencer@uni-muenster.de


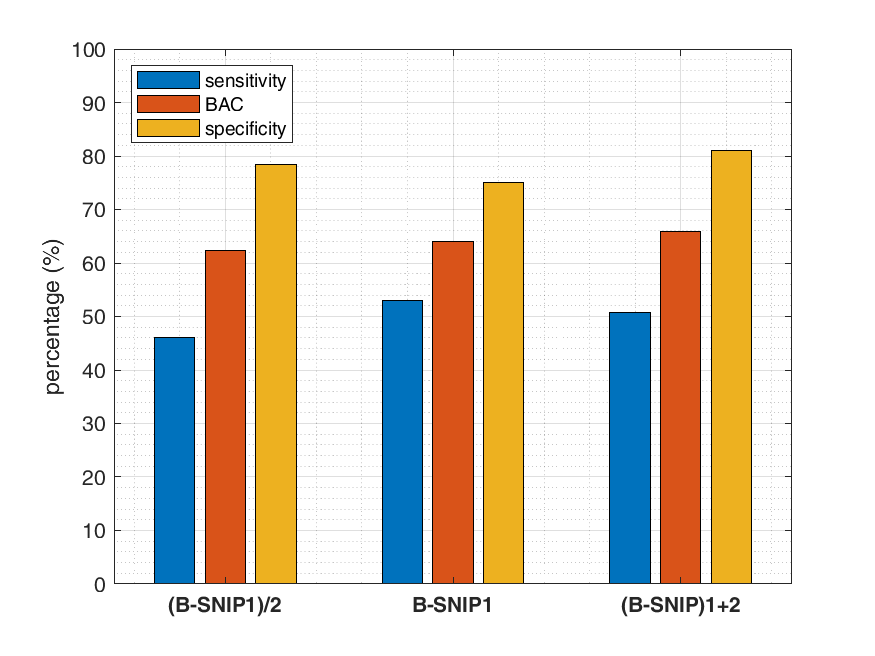


#

**Fig. S1.** Dependency of model parameters from sample size.

Figure displays the effect of sample size on model performance (therefore, models were trained and internally validated in half of the B-SNIP1 [(B-SNIP)/2)] and in the combined B-SNIP1 and B-SNIP2 [(B-SNIP)1+2)] samples). BAC=Balanced accuracy.

|  | **Original scores** | | **Converted scores** | |
| --- | --- | --- | --- | --- |
|  | **SANS** | **SAPS** | **PANSS negative** | **PANSS positive** |
| Controls | 0.64 (1.93) | 0.12 (0.56) | 7.34 (0.65) | 11.22 (0.14) |
| Psychosis Probands | 18.12 (17.82) | 8.65 (12.86) | 13.21 (5.99) | 13.43 (3.33) |
| MDwoP | 11.20 (12.84) | 0.60 (1.39) | 10.89 (4.32) | 11.34 (0.36) |
| BPwoP | 11.28 (12.42) | 1.08 (2.91) | 10.91 (4.18) | 11.47 (0.75) |

**Table S1.** Psychosis symptoms (conversion from SANS/SAPS to PANSS scores for the FOR2107 study)

**Abbreviations:** FOR2107=DFG Forschergruppe 2107, MDwoP=probands with major depression without psychosis, BPwoP=bipolar probands without psychosis, SAPS=Scale for Assessment of Positive Symptoms and SANS=Scale for Assessment of Negative symptoms ^1^, PANSS=Positive And Negative Syndrome Scale ^2^. Conversion was estimated using the computations from Van Erp and colleagues ^3^ using SANS and SAPS composite/total scores.

| **Study** | **Total score^a^** | **No depression/**  **remission** | **Mild depression** | **Moderate depression** | **Severe depression** |
| --- | --- | --- | --- | --- | --- |
| **B-SNIP1** |  |  |  |  |  |
| Controls |  |  |  |  |  |
| Psychosis probands | 10.74 (9.32) | 50.6% | 26.7% | 21.2% | 1.5% |
| **B-SNIP2** |  |  |  |  |  |
| Controls |  |  |  |  |  |
| Psychosis probands | 11.41 (10.24) | 47.0% | 28.9% | 20.6% | 3.6% |
| **PARDIP** |  |  |  |  |  |
| Controls |  |  |  |  |  |
| BPwP | 17.86 (12.46) | 34.7% | 16.3% | 40.8% | 8.2% |
| BPwoP | 17.15 (11.05) | 23.5% | 35.3% | 35.3% | 5.9% |
| **FOR2107** |  |  |  |  |  |
| Controls | 2.65 (3.42) |  |  |  |  |
| Psychosis Probands | 12.11 (8.07) |  |  |  |  |
| MDwoP | 11.95 (9.65) |  |  |  |  |
| BPwoP | 11.88 (13.45) |  |  |  |  |
| **PRONIA** |  |  |  |  |  |
| Controls | 3.07 (2.56) | 100% |  |  |  |
| ROD | 22.71 (11.36) | 21.4% | 21.4% | 28.6% | 28.6% |
| CHR | 31.06 (8.98) | 0.0% | 0.0% | 43.8% | 56.3% |
| ROP | 28.10 (16.39) | 20.0% | 0.0% | 30.0% | 50.0% |

**Table S2.** Depression (gradation estimation)

**Abbreviations:** B-SNIP=Bipolar-Schizophrenia Network on Intermediate Phenotypes, PARDIP= Psychosis and Affective Research Domains and Intermediate Phenotypes, BPwP=bipolar probands with psychosis, BPwoP=bipolar probands without psychosis, FOR2107=DFG Forschergruppe 2107, MDwoP=probands with major depression without psychosis, PRONIA=Personalised Prognostic Tools for Early Psychosis Management, ROD=recent-onset depression probands, CHR=clinical-high-risk- for psychosis probands, ROP=recent-onset-psychosis probands. **Further explanations: ^a^**Depressive symptoms were estimated using the following measures: B-SNIP1, B-SNIP2, PARDIP=Montgomery–Åsberg Depression Rating Scale (MADRS; ^4^); FOR2107=Original Beck Depression Inventory, 1978 version ^5^; PRONIA=Beck Depression Inventory-II (BDI-II; ^6^). Severity gradation was calculated according to MADRS: ^7^. BDI: ^6^. No severity gradation could be computed for the original Beck Depression Inventory used in the FOR 2107 sample.

|  | **Classifier** | **Specified parameters** | **Balancing method** | **PCA components** |
| --- | --- | --- | --- | --- |
| **Overall best configuration** | Support vector machine | C=1, linear kernel | Undersampling majority class | 3 |
| **Outer fold 1** | Support vector machine | C=0.5, rbf kernel | SMOTE | disabled |
| **Outer fold 2** | Support vector machine | C=0.1, linear kernel | Undersampling majority class | 2 |
| **Outer fold 3** | Support vector machine | C=1, linear kernel | Undersampling majority class | 3 |

**Table S3.** Specifications of the best machine learning model

|  | **TP** | **FP** | **TN** | **FN** | **BAC** | **Sensitivity** | **Specificity** | **PPV** | **NPV** | **P-value** |
| --- | --- | --- | --- | --- | --- | --- | --- | --- | --- | --- |
| **B-SNIP1** | | | | | | | | | | |
| Fold 1 | 122 | 32 | 72 | 101 | 61.97 | 54.71 | 69.23 | 79.22 | 41.62 |  |
| Fold 2 | 124 | 22 | 67 | 113 | 63.80 | 52.32 | 75.28 | 84.93 | 37.22 |  |
| Fold 3 | 111 | 22 | 90 | 103 | 66.11 | 51.87 | 80.36 | 83.46 | 46.63 |  |
| **Mean** |  |  |  |  | **63.96** | **52.97** | **74.96** | **82.54** | **41.82** | **<.001** |
| **B-SNIP2 (external validation-1)** | | | | | | | | | | |
| Psychosis probands vs. Controls | 373 | 75 | 214 | 293 | **65.03** | 56.01 | 74.05 | 83.26 | 42.21 |  |
| **PARDIP (external validation-2)** | | | | | | | | | | |
| BPwP vs. Controls | 30 | 26 | 44 | 14 | **65.52** | 68.18 | 62.86 | 53.57 | 75.86 |  |
| **FOR2107 (external validation-3)** | | | | | | | | | | |
| Psychosis probands vs. Controls | 22 | 19 | 53 | 29 | **58.37** | 43.14 | 73.61 | 53.66 | 64.63 |  |

**Table S4.** Model results for the comparison of chronic psychosis probands vs. controls for B-SNIP1 and external validation samples B-SNIP2, PARDIP, and FOR2107

**Abbreviations:** B-SNIP=Bipolar-Schizophrenia Network on Intermediate Phenotypes, TP=true positive findings, FP=false positive findings, TN=true negative findings, FN=false negative findings, BAC=balanced accuracy score, PPV=positive predictive value, NPV=negative predictive value, FOR2107=DFG Forschergruppe 2107.
**Further Explanations:** P-value is indicated for BAC score as this metric was used to identify the best performing model.

|  | **TP** | **FP** | **TN** | **FN** | **BAC** | **Sensitivity** | **Specificity** | **PPV** | **NPV** |
| --- | --- | --- | --- | --- | --- | --- | --- | --- | --- |
| **schizophrenia probands vs. schizoaffective probands** | | | | | | | | | |
| Fold 1 | 47 | 19 | 33 | 49 | 56.21 | 48.96 | 63.46 | 71.21 | 40.24 |
| Fold 2 | 45 | 26 | 34 | 43 | 53.90 | 51.14 | 56.67 | 63.38 | 44.16 |
| Fold 3 | 37 | 33 | 33 | 44 | 47.84 | 45.68 | 50.00 | 52.86 | 42.86 |
| **Mean** |  |  |  |  | **52.65** | **48.59** | **56.71** | **62.48** | **42.42** |
| **schizophrenia probands vs. bipolar probands** | | | | | | | | | |
| Fold 1 | 39 | 23 | 50 | 54 | 55.21 | 41.94 | 68.49 | 62.90 | 48.08 |
| Fold 2 | 37 | 35 | 43 | 50 | 48.83 | 42.53 | 55.13 | 51.39 | 46.24 |
| Fold 3 | 27 | 20 | 60 | 58 | 53.38 | 31.76 | 75.00 | 57.45 | 50.85 |
| **Mean** |  |  |  |  | **52.48** | **38.74** | **66.21** | **57.25** | **48.39** |
| **schizoaffective probands vs. bipolar probands** | | | | | | | | | |
| Fold 1 | 29 | 46 | 36 | 26 | 48.31 | 52.73 | 43.90 | 38.67 | 58.06 |
| Fold 2 | 21 | 33 | 46 | 36 | 47.54 | 36.84 | 58.23 | 38.89 | 56.10 |
| Fold 3 | 50 | 43 | 27 | 16 | 57.16 | 75.76 | 38.57 | 53.76 | 62.79 |
| **Mean** |  |  |  |  | **51.00** | **55.11** | **46.90** | **43.77** | **58.98** |

**Table S5.** Model results for individual psychosis proband groups in the B-SNIP1 sample

**Abbreviations:** B-SNIP=Bipolar-Schizophrenia Network on Intermediate Phenotypes, TP=true positive findings, FP=false positive findings, TN=true negative findings, FN=false negative findings, BAC=balanced accuracy score, PPV=positive predictive value, NPV=negative predictive value.
**Further Explanations:** P-value is indicated for BAC score as this metric was used to identify the best performing mode.

|  | **TP** | **FP** | **TN** | **FN** | **BAC** | **Sensitivity** | **Specificity** | **PPV** | **NPV** |
| --- | --- | --- | --- | --- | --- | --- | --- | --- | --- |
| Fold 1 | 53 | 17 | 38 | 55 | 59.08 | 49.07 | 69.09 | 75.71 | 40.86 |
| Fold 2 | 50 | 7 | 46 | 60 | 66.12 | 45.45 | 86.79 | 87.72 | 43.40 |
| Fold 3 | 52 | 9 | 35 | 67 | 61.62 | 43.70 | 79.55 | 85.25 | 34.31 |
| **Mean** |  |  |  |  | **62.28** | **46.08** | **78.48** | **82.89** | **39.52** |

**Table S6.** Model results for 50% of the B-SNIP1 sample

**Abbreviations:** B-SNIP=Bipolar-Schizophrenia Network on Intermediate Phenotypes, TP=true positive findings, FP=false positive findings, TN=true negative findings, FN=false negative findings, BAC=balanced accuracy score, PPV=positive predictive value, NPV=negative predictive value.
**Further Explanations:** P-value is indicated for BAC score as this metric was used to identify the best performing model.

|  | **TP** | **FP** | **TN** | **FN** | **BAC** | **Sensitivity** | **Specificity** | **PPV** | **NPV** |
| --- | --- | --- | --- | --- | --- | --- | --- | --- | --- |
| Fold 1 | 246 | 43 | 154 | 223 | 65.31 | 52.45 | 78.17 | 85.12 | 40.85 |
| Fold 2 | 210 | 30 | 163 | 263 | 64.43 | 44.40 | 84.46 | 87.50 | 38.26 |
| Fold 3 | 255 | 41 | 166 | 204 | 67.87 | 55.56 | 80.19 | 86.15 | 44.86 |
| **Mean** |  |  |  |  | **65.87** | **50.80** | **80.94** | **86.26** | **41.33** |

**Table S7.** Model results for combined B-SNIP1 and B-SNIP2 samples

**Abbreviations:** B-SNIP=Bipolar-Schizophrenia Network on Intermediate Phenotypes, TP=true positive findings, FP=false positive findings, TN=true negative findings, FN=false negative findings, BAC=balanced accuracy score, PPV=positive predictive value, NPV=negative predictive value.
**Further Explanations:** P-value is indicated for BAC score as this metric was used to identify the best performing model.

| **Study** | **Group** | **SPEM variables** | | | |
| --- | --- | --- | --- | --- | --- |
|  |  | Predictive maintenance gain | Early maintenance gain | Initial eye acceleration | Eye latency |
| **B-SNIP1** | Psychosis probands | -.05 (n.s.) | -.11 (p=.008) | -.08 (n.s.) | .08 (n.s.) |
| **B-SNIP2** | Psychosis probands | -.08 (n.s.) | -.09 (p=.03) | .02 (n.s.) | .03 (n.s.) |
| **PARDIP** | BPwP | .11 (n.s.) | .06 (n.s.) | -.05 (n.s.) | -.02 (n.s.) |
| **FOR2107** | Psychosis probands | -.05 (n.s.) | -.11 (n.s.) | -.05 (n.s.) | .17 (n.s.) |
| **PRONIA** | ROP | .07 (n.s.) | .33 (n.s.) | .20 (n.s.) | .07 (n.s.) |

**Table S8.** Correlations between SPEM variables and chlorpromazine equivalents

**Abbreviations:** B-SNIP=Bipolar-Schizophrenia Network on Intermediate Phenotypes**,** PARDIP= Psychosis and Affective Research Domains and Intermediate Phenotypes, BPwP=bipolar probands with psychosis, BPwoP=bipolar probands without psychosis**,** PRONIA=Personalised Prognostic Tools for Early Psychosis Management, ROD=recent-onset depression probands, CHR=clinical-high-risk- for psychosis probands, ROP=recent-onset-psychosis probands, n.s.=non-significant (p values were shown Bonferroni-Holm corrected).

| **Group** | **SPEM variables** | | | |
| --- | --- | --- | --- | --- |
|  | Predictive maintenance gain | Early maintenance gain | Initial eye acceleration | Eye latency |
| **Psychosis probands** | .08 (p=.006) | .13 (p=.004) | .11 (p=.004) | -.04 (n.s.) |
| **Healthy controls** | .10 (n.s.) | .09 (n.s.) | .08 (n.s.) | -.05 (n.s.) |

**Table S9.** Correlations between SPEM variables and Wide Range Achievement Test 4 in the B-SNIP1 sample

**Abbreviations:** B-SNIP=Bipolar-Schizophrenia Network on Intermediate Phenotypes**,** n.s.=non-significant (p values were shown Bonferroni-Holm corrected).

| **Group** | **SPEM variables** | | | |
| --- | --- | --- | --- | --- |
|  | Predictive maintenance gain | Early maintenance gain | Initial eye acceleration | Eye latency |
| **Psychosis probands** | .14 (p=.004) | .19 (p=.004) | .15 (p=.004) | -.06 (p=.01) |
| **Healthy controls** | .04 (n.s.) | .09 (n.s.) | .05 (n.s.) | -.02 (n.s.) |

# Table S10. Correlations between SPEM variables and z-scores of the Brief assessment of cognition in schizophrenia in the B-SNIP1 sample

**Abbreviations:** B-SNIP=Bipolar-Schizophrenia Network on Intermediate Phenotypes**,** n.s.=non-significant (p values were shown Bonferroni-Holm corrected).

| **SPEM variable** | **ANOVA** | **Post-hoc (Bonferroni-corrected alpha-level)** |
| --- | --- | --- |
| Predictive maintenance gain | F(4,751)=2.44, p=.05 | all comparisons were non-significant (p≥.20) |
| Early maintenance gain | F(4,750)=2.66, p=.03 | B-SNIP1 > FOR2107 p=.02, all other comparisons were non-significant (p≥.48) |
| Initial eye acceleration | F(4,747)=7.51, p<.001 | B-SNIP1 < FOR2107 p<.001  B-SNIP1 < PRONIA p=.01  B-SNIP2 < FOR2107 p=.001  B-SNIP2 < PRONIA p=.02  PARDIP < FOR2107 p<.001  PARDIP < PRONIA p=.005  all other comparisons were non-significant (p=1.00) |
| Eye latency | F(4,748)=6.74, p<.001 | B-SNIP1 > FOR2107 p=.02  B-SNIP2 > FOR2107 p<.001  PARDIP > FOR2107 p<.001  all other comparisons were non-significant (p≥.11) |

# Table S11. Differences between laboratories/recording devices in SPEM variables of healthy controls

|  | **TP** | **FP** | **TN** | **FN** | **BAC** | **Sensitivity** | **Specificity** | **PPV** | **NPV** |
| --- | --- | --- | --- | --- | --- | --- | --- | --- | --- |
| Fold 1 | 94 | 21 | 83 | 129 | 60.98 | 42.15 | 79.81 | 81.74 | 39.15 |
| Fold 2 | 138 | 30 | 59 | 99 | 62.26 | 58.23 | 66.29 | 82.14 | 37.34 |
| Fold 3 | 103 | 26 | 86 | 111 | 62.46 | 48.10 | 76.79 | 79.84 | 43.65 |
| **Mean** |  |  |  |  | **61.90** | **49.50** | **74.30** | **81.24** | **40.05** |

**Table S12.** Model results B-SNIP1 sample (comparison psychosis probands vs. controls, predictors: predictive maintenance gain, early maintenance gain)

**Abbreviations:** B-SNIP=Bipolar-Schizophrenia Network on Intermediate Phenotypes, TP=true positive findings, FP=false positive findings, TN=true negative findings, FN=false negative findings, BAC=balanced accuracy score, PPV=positive predictive value, NPV=negative predictive value.
**Further Explanations:** P-value is indicated for BAC score as this metric was used to identify the best performing model.

**References**

1. Andreasen, N. C. The Scale for the Assessment of Negative Symptoms (SANS): Conceptual and Theoretical Foundations. *Br. J. Psychiatry* **155**, 49–52 (1989).

2. Kay, S. R., Fiszbein, A. & Opler, L. A. The positive and negative syndrome scale (PANSS) for schizophrenia. *Schizophr. Bull.* **13**, 261–276 (1987).

3. Van Erp, T. G. M. *et al.* Converting positive and negative symptom scores between PANSS and SAPS/SANS. *Schizophr. Res.* **152**, 289–294 (2014).

4. Montgomery, S. A. & Åsberg, M. A new depression scale designed to be sensitive to change. *Br. J. Psychiatry* **134**, 382–389 (1979).

5. Beck, A. T. & Steer, R. A. Internal consistencies of the original and revised Beck Depression Inventory. *J. Clin. Psychol.* **40**, 1365–1367 (1984).

6. Beck, A. T., Steer, R. A. & Brown, G. K. *Manual for Beck Depression Inventory-II*. (San Antonio, TX: Psychological Corporation, 1996).

7. Müller, M. J., Szegedi, A., Wetzel, H. & Benkert, O. Moderate and severe depression: Gradations for the Montgomery–Åsberg Depression Rating Scale. *J. Affect. Disord.* **60**, 137–140 (2000).
